# Expression of concern: Reduced interleukin-38 in non-small cell lung cancer is associated with tumour progression

**DOI:** 10.1098/rsob.200152

**Published:** 2020-06-10

**Authors:** Feng Wang, Weihua Zhang, Tianfeng Wu, Heying Chu

*Open Biol.* **8**, 180132. (Published online 31 October 2018) (doi:10.1098/rsob.180132)

Following the publication of Reduced interleukin-38 in non-small cell lung cancer is associated with tumour progression *Open Biol*. 8: 180132. (Published online 31 October 2018), the Editorial team have very serious concerns regarding the validity of the data used in this study.

We are issuing an expression of concern here and investigating this as a matter of urgency; we will notify readers as to the results of our investigation as soon as possible.

